# Preferences of women with a vulnerable health status towards nudging for adequate pregnancy preparation as investment in health of future generations: a qualitative study

**DOI:** 10.1186/s12884-022-04887-6

**Published:** 2022-07-13

**Authors:** Sharissa M. Smith, Rianne M. J. J. van der Kleij, Babette Bais, Maartje H. N. Schermer, Hafez Ismaili M’hamdi, Régine P. M. Steegers-Theunissen

**Affiliations:** 1 Department of Obstetrics and Gynaecology, Erasmus MC, University Medical Centre, Rotterdam, the Netherlands; 2 Department of Public Health and Primary Care, Leiden University Medical Centre, Leiden, the Netherlands; 3 Department of Medical Ethics, Philosophy and History of Medicine, Erasmus MC, University Medical Centre, Rotterdam, the Netherlands

**Keywords:** Preconception care, Nudge, Socioeconomic factors, Lifestyle, Ethics, Rewards, Life course

## Abstract

**Background:**

Women with a vulnerable health status, as determined by a low socioeconomic status and poor lifestyle behaviours, are at risk for adverse pregnancy outcomes. Offering tailored preconception lifestyle care can significantly help to improve pregnancy outcomes. We hypothesize that so-called ‘nudges’ can be a successful way of increasing the uptake of preconception lifestyle care. A nudge is a behavioural intervention that supports healthy choices by making them easier to choose. Nudging, however, raises many moral questions. Effectiveness and respect for autonomy are, among other criteria, required for a nudge to be morally permissible. In general, the target group knows best what they find permissible and what would motivate them to change their lifestyle. Therefore, this study – conducted in women with a vulnerable health status – aimed to identify their preferences towards a nudge, provided via a mobile application that aims to help them adopt healthy lifestyle behaviours by offering rewards.

**Methods:**

We conducted semi-structured interviews with twelve women with a vulnerable health status. A framework approach was used to analyse the data. A thematic content analysis was conducted on five themes: (1) “Usefulness of an app as an integral information source”, (2) “Permissibility and effects of offering rewards”, (3) “Preferences regarding content”, (4) “Preferences regarding type of rewards and system of allocation”, and (5) “Barriers”.

**Results:**

Of the 12 participants, 11 deemed an app as integral information source concerning the preconception period useful. None of the participants objected to being nudged i.e., being rewarded for healthy behaviour. All participants stated that they would like the app to contain information on healthy nutrition and 8 participants wanted to know how to get pregnant quickly. Furthermore, participants stated that the freedom to choose the timing and content of the reward would increase the probability of successful behavioural change, and having to pay or contact a healthcare provider to access the app may prevent women using the app.

**Conclusions:**

These insights into the preferences of women with a vulnerable health status towards nudging will inform the design of an effective app-based nudge. This may help to improve prepregnancy health as investment in health of current and future generations.

## Background

In the Netherlands, women with a low socioeconomic status (SES) are more likely to have adverse pregnancy outcomes than women with a higher SES [1–3]. Low SES is associated with chronic stress, affecting maternal and offspring health during the life course [4, 5]. Furthermore, low SES is associated with poor health and unhealthy lifestyle behaviours such as poor diet, smoking and sedentary lifestyle. For instance, Thornton et al. found that women in low SES neighbourhoods had poorer diets than women in high SES neighbourhoods, with an odds ratio for two or more servings of vegetable intake of 0.33 (0.23–0.45) [6]. Stringhini et al. found that people with a low SES were more likely to smoke than people with a high SES (29.7% vs. 10.1%) [7]. These behaviours contribute to the higher incidence of adverse pregnancy of these women as well [8].

Adequately preparing for pregnancy in the period preceding conception, the preconception period, positively influences pregnancy outcomes [9, 10]. Offering ‘preconception care’ (PCC) and encouraging women with a low SES in particular to adequately prepare for pregnancy can therefore especially aid at decreasing adverse pregnancy outcomes. However, these women are less likely to participate in PCC because they are often not aware of the existence and importance of PCC [11]. Furthermore, this is a difficult group to reach and to motivate to participate in PCC [12].

However, there are opportunities to encourage these women to participate in PCC. Smartphones are widely available, even for women with a low SES [13]. Offering PCC as a mobile Health (mHealth) application (app) can reduce barriers and encourage these women to prepare for pregnancy. The mHealth app www.SmarterPregnancy.co.uk (www.SlimmerZwanger.nl) for example, provides evidence-based coaching that aims to help women who wish to become pregnant adopt and maintain healthy lifestyle behaviours [14, 15]. Women fill out a questionnaire concerning their dietary habits, sleep, stress, exercise and alcohol and tobacco use, and subsequently receive personalised coaching through the app and per email, based on their answers. Smarter Pregnancy has shown to improve lifestyle behaviours up to 30% and increases the chance of pregnancy in couples who received fertility treatment up to 50%, even in women who live in deprived neighbourhoods [14, 15]. However, changing lifestyle and adopting healthy behaviours is challenging for all, but in particular for women who live in the most stressful situations in deprived neighbourhoods [14]. Chronic exposure to stressors impedes their abilities to adopt healthy lifestyle behaviours. Therefore, these women should receive more support to be successful at improving their lifestyle and subsequent (reproductive) health.

An option to increase the effectivity of lifestyle support is the use of so-called ‘nudges’. The definition of a nudge is *“any aspect of the choice architecture that alters people’s behaviour in a predictable way without forbidding any options or significantly changing their economic incentives” *[15]. In other words, a nudge is an intervention that alters people’s behaviour and facilitates healthy choices, by influencing the process of choice-making in a noncoercive, nonobstructive manner [15, 16]. In the future, we plan to add rewards (e.g. beauty or baby products) for healthy behaviour to an app, similar to Smarter Pregnancy, to develop an app-based nudge as a preconceptional lifestyle intervention. Using rewards as nudges is a fairly new approach and has to adhere to certain conditions to still count as a nudge. For example, by definition, a nudge may not significantly change economic incentives. Therefore, the value of the rewards must be limited to prevent coercion. An app-based nudge will facilitate choosing healthy behaviour by making it easier and more fun, thus supporting women’s efforts to improve their health by adopting healthy lifestyle behaviours. Programs that use rewards to nudge participants and encourage commitment are called loyalty programs [17].

Nudging, however, can also lead to possible harm [18]. For example, providing a reward for gym classes may lead to an initial increase of participants. Attendance may drop however, when the reward is taken away, which could lead to lower attendance than before the intervention started. To avoid such pitfalls, we have previously designed an ethical framework detailing which conditions a morally permissible nudge must satisfy. The criteria in our framework are subdivided under the four ethical principles of Beauchamp & Childress [19]; 1. Respect for Autonomy, 2. Beneficence, 3. Non-Maleficence & 4. Justice. We have chosen these principles to support ethical considerations, because the principlist approach allows careful balancing between improving the health and wellbeing of vulnerable women (and their offspring), and the duty to respect these women and treat them as free and equal persons.

One of the most important criteria we identified is that, to be effective, a nudge has to be aligned with the preferences of the target group and therefore should be developed in cocreation [20–22]. After all, the target group often knows what would be most effective for them. Moreover, there is discussion about possible infringements on freedom of choice (i.e. insufficient respect for autonomy) by nudges [15]. To address these issues and to support the design of an app-based nudge for women who are vulnerable for adverse pregnancy outcomes, we conducted a qualitative study to identify the preferences and opinions of this specific target group towards a nudge that aims to help them to adopt healthy lifestyle behaviours.

## Methods

### Recruitment

**Fig. 1:**
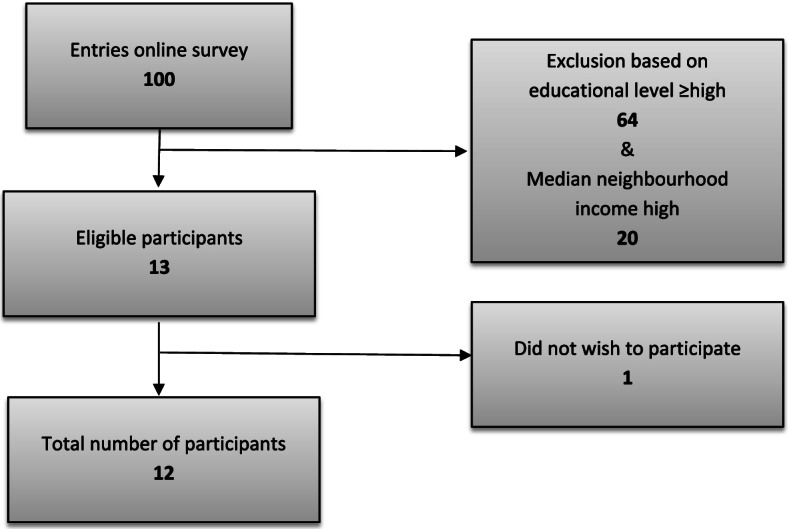
Inclusion flowchart

The recruitment strategy included distribution of flyers and posters, and recruitment through social media posts between November 2019 and January 2020. Women between 18 and 45 years of age, with children or those who wish to have a child in the future, were asked to fill out a short online survey. The survey included questions on 1. age, 2. ZIP code, 3. educational level, 4. (previous) pregnancies, and 5. if they wished to become pregnant, and if so, in what time frame (actively trying to conceive, planning to try to conceive within 1 year, within three years 3 years or after 3 years/not yet actively planning). Question 4 and 5 were implemented in the survey to ensure selection of women who either already had children or wished to have at least one child in their lifetime, as they are the prospective users of the app-based nudge. A total of 100 women filled in the online survey. Based on the questionnaire data, 12 women were selected for an interview (see Fig. 1 and Table 1). We selected women with a low to intermediate educational level, who live in a neighbourhood with a low median to middle median household income, which we have used as proxy for a low SES [23]. Women who finished the interview received a €35 (≈$42) voucher to compensate for their time investment.

**Table 1:** Baseline characteristics (*n* = 12)

Demographics		Participants
Age in years, median (range)		30.5 (18–42)
Educational level^b^	Low	6 (50%)
	Intermediate	6 (50%)
Median income neighbourhood^a^	Low	7 (58%)
	Middle	5 (41.7%)
Parity	Nulliparous	5 (41.7%)
	1	5 (41.7%)
	≥ 2	2 (16.7%)

^a^Educational level [24]. The Dutch educational levels are subdivided as follows; Low: prevocational education, selective secondary education or lower. Intermediate: vocational education. High: bachelor’s degree, master’s degree or higher

^b^The median household income of a neighbourhood is determined by the distribution of household income of all households in the country. The median income of a neighbourhood is equal to the middle income if all households are ranked from low to high. Low: < €21.000 ($24.800), Middle: €21.000—€26.800 ($24.800—$31.700), Middle-High: €26.800—€34.600 ($31.700—$40.900), High > €34.600 ($31.700)

### Data collection

Semi-structured interviews were conducted in January 2020 by SS and were carried out using a topic list that consisted of five topics; (1) the participants’ opinions on the usefulness of an app as an integral source of information that helps them adequately prepare for pregnancy, (2) the permissibility and expected effects of offering rewards for healthy behaviour, (3) their preferences regarding the content of the app, (4) their preferences regarding the type of rewards and system of allocation, and (5) possible barriers towards the use of the app. The system of allocation refers to the way in which actions are connected to rewards. The topic list was compiled based on the information required to design the app. First, we wanted to know if the target group would want an app as source of information at all. Second, it was important whether they considered it permissible to be rewarded for healthy behaviour. If so, topic III, IV, and V provided preferences regarding the direct design of the app. On January 31^st^ 2020, saturation of data was reached and the online questionnaire was closed.

### Data analysis

For data analysis, we have used a framework approach based on the work of Gale et al. [25]. The approach consisted of 5 stages; 1. Transcription, 2. Familiarisation with the data, 3. Coding and developing a framework 4. Charting the data into a framework matrix and 5. Interpretation of the data.

#### Stage 1, transcription

All interviews were audio-recorded and transcribed verbatim for analysis by SS. The transcripts were anonymised by not transcribing mentioned names and replacing the name of the participant with a letter.

#### Stage 2, familiarisation with the data

All transcripts were uploaded into NVivo11 software for Windows10 and were reread and annotated by SS.

#### Stage 3, coding and developing a framework

During transcription, rereading and annotating, SS extracted five themes from the interviews, which were confirmed by HI and RK. Subsequently, SS set up an initial coding scheme by adding child nodes that were extracted from the data in the same manner. During coding, this scheme was expanded and adjusted through an iterative process. The coding scheme was validated independently by HI, by reading all twelve interviews and reviewing the coding scheme.

#### Stage 4, charting the data into a framework matrix

SS created an overview of the coding scheme and the interview outcomes, using Microsoft Excel. Each subject of the coding scheme was attributed a symbol (+ , ± , or -) that marked whether or not the participant respectively agreed, was ambiguous or disagreed on a subject. If a topic was not mentioned in the interview, the box was left empty. In this Excel sheet, citations that were representative of certain findings were added and translated to English.

#### Stage 5, interpretation of the data

The Excel sheet was studied in order to identify the most often occurring opinions and preferences of the participants. Furthermore, notable highlights, such as opinions that strongly deviated from the majority, were also identified.

## Results

### Study participant’s characteristics

Our study group consisted of twelve women, aged between 18 and 42 years (median 30.5 years), with a low to intermediate educational level (see Table 1). Eleven participants had a non-western background and seven of them had at least one child. Six women lived in a neighbourhood with a low median household income, one woman lived in a neighbourhood with a low to middle median household income, and five women lived in a neighbourhood with a middle median household income.

### Preferences and opinions

The results of the interviews were subdivided according to the themes of the topic list. See Table 2 for an overview of the results.

**Table 2:** Overview of the results

Themes	Outcomes		ɳ	Summary
**Usefulness the app as integral information source**	App deemed useful		10/12	• Integral source of information is very welcome • Amount of available information is overwhelming • Available information is scattered
	App deemed not useful		2/12	• Prefer to follow own rules
**Permissibility & effects of rewards**	Rewards deemed permissible		12/12	• Rewards form additional source of motivation
	Doubts on permissibility		2/12	• Rewards for healthy behaviour might feel uneasy
	Rewards deemed effective		11/12	• Rewards may spike increased interest in the app • Earning points feels like a game/challenge
	Doubts on effectiveness		3/12	• Persons who use the app are already motivated
**Wishes for content of the app**	Desired information		-	• Nutrition, exercise, preconception period, fertility • Effects of (abstaining from) tobacco/alcohol/drugs • Rationale behind the given advice • Information on pregnancy and postpartum period
	Desired features		-	• User friendly dashboard • Personalised advice • In-app communication with peers
**Preferences rewards & allocation system**	Desired rewards		-	• Baby products, books, sportswear, pregnancy vitamins, luxury goods, healthy food
	System of allocation	Points	5/12	• Freedom to choose when/how to spend points
		Goals	3/12	• Saving points lacks an endpoint
**Barriers**	Anticipated barriers		-	• Payment for the app (≥ €0,01) • Asking healthcare provider for access • Not considering themselves part of the target group
	Not considered barriers		-	• Stigmatisation • Shame

#### Usefulness of an app as integral information source

##### *Usefulness of an app as integral source of information for pregnancy preparation*

One-third of the participants mentioned being overwhelmed by the vast amount of pregnancy related information available on the internet. Two participants mentioned that they felt the available information was scattered, incomplete, or subjective. Ten participants agreed that one integral, reliable source of information regarding pregnancy and pregnancy preparation would be welcome. *“It was just too much, just too much. Of course there are many ways to get pregnant, but I could not find the best way or the fastest way to get pregnant, or determine what was most important. I couldn't really filter that out.” (Interview C, 30 y.o., two children.)*

One participant stated that, for her, the app would not be useful because she prefers to follow her own rules. *“I don't really need such an app. I actually prefer to follow my own rules.” (Interview J, 33 y.o., three children.)*

#### Permissibility and effects of offering rewards

##### *Permissibility of offering rewards for pregnancy preparation*

None of the participants objected to receiving rewards for adopting lifestyle behaviours. Two participants did not object, but did express some doubt about whether or not women should receive rewards for healthy behaviour, stating it may feel uneasy being rewarded for something you should (be able to) do for yourself. *“It’s not like it’s supposed to be, but [receiving a reward] would be very nice and I think it uh, especially people having a hard time would really appreciate it because you can get something positive out of it.” (Interview C, 30 y.o., two children.)*

Most participants viewed the rewards as an additional source of motivation that would add up to the motivation already present in the mothers-to-be. *“Fine right? You actually do it more for yourself. That’s how I see it. And if you get a reward in return, yes, why not?” (Interview A, 39 y.o., one child.)*

Despite the fact that she does not object to rewarding women for healthy behaviour, one participant stated that she would not use the app. *“[I wouldn't use the app] because I'd rather achieve my goals in a different way. I would want to check off my goals, but not receive a reward. That's still a bit … It would feel like I’m not disciplined enough and therefore need an app.” (Interview H, 20 y.o., nulliparous.)*

##### Expected effects of offering rewards for pregnancy preparation

Eleven of the participants mentioned that offering rewards for adopting healthy lifestyle behaviours would be an effective strategy. More than half of the participants mentioned that the possibility of earning rewards would lead to an increased interest in the app amongst their friends. *“If I have the app, I would motivate my friends too. If I can show them, I think they'll get motivated too. I’m getting points and that is how I am rewarded. I feel appreciated, I receive this, I receive that….” (Interview G, 22 y.o. nulliparous.)*

Furthermore, one participant mentioned that she would consider it a challenge to earn a reward. This game-like feature would amuse her and keep her focused on making lifestyle changes. *“It depends on your point of view, but I think that [rewards] are effective. I think it will be much more fun. Yes, the challenge in it. You are going to challenge yourself to get a reward.” (Interview E, 36 y.o., one child.)*

Three participants mentioned that women who would be interested in using the app would already have some motivation to make lifestyle changes, which caused them to doubt the effect of rewards on lifestyle behaviour. “*If you have such an app, it already means you want to go for it [prepare for pregnancy]. In that case, the app may not make a difference. If I wasn’t motivated, I wouldn’t have the app at all.” (Interview I, 19 y.o., nulliparous.)*

#### Preferences regarding the content of the app

##### *Preferences regarding informational content of the app*

Participants mentioned to appreciate receiving information on the following topics; nutrition, exercise, the preconception period, fertility, the effects of (abstaining from) alcohol, tobacco and drugs, the development of the baby, and the postpartum period.

All participants mentioned advice on nutrition as an essential topic. This included advice on what a healthy diet consists of, what women should and should not consume, and which supplements to take before and during pregnancy. *“What, then, is healthy eating? Because there is healthy eating and healthy eating. And … Yes, which vitamins you should take, or what you should not do, for example.” (Interview L, 31 y.o., one child.)*

Furthermore, four participants were explicitly interested in how they could increase their level of fitness, which forms of exercise are recommended, and which types of exercise to avoid. Two-thirds of the participants stated that they would want to get pregnant fast and therefore would like to receive information to help them reach the goal of getting pregnant as quickly as possible.

During the interviews, the participants received information about the preconception period and the possibility of influencing the course of their pregnancy before conception by improving their lifestyle behaviours in the preconception period. Seven participants were surprised by this information and stated that this should be more widely known. Therefore, this information should be included in the app. *“But I did not really think about that it may have helped to quit smoking or … I did not look up that information. Let me put it this way. It was… I want a child, and you hear about iron or vitamin or whatever you need. So my first reaction was just to take folic acid. I did not look at all the other things that could also play a role [in how to quickly become pregnant].” (Interview E, 36 y.o., one child.)*

Abstaining from alcohol, tobacco, and drugs in pregnancy was often mentioned as standard advice which is known to everyone. Nevertheless, three participants mentioned that they found it hard to abstain from tobacco or knew women who had continued smoking during their pregnancy, despite the common knowledge about the detrimental effects.

Two participants stated that they would want to know the rationale behind the advice because it would help them take the advice more seriously. *“I would like to know the background of the advice so you can, uh, really take seriously the effect it [lifestyle] can have [on becoming pregnant or the course of the pregnancy].” (Interview H, 20 y.o., nulliparous.)*

Furthermore, four participants specifically stated that they wished the app to continue to provide information in pregnancy and the postpartum period. For example, they would like to receive information about the development of their baby outside of the womb, how to increase the chance of breastfeeding successfully, and how to healthily lose pregnancy weight.

##### *Preferences regarding the features of the app*

When inquired about which properties they would like the app to have, the participants mentioned the following; (1) a clear and user-friendly dashboard in which they can keep track of their goals and improvements, (2) personalised advice, based on their personal lifestyle behaviours, BMI and level of fitness, and (3) a way to communicate with their peers through, for example, a chat or forum, about their (future) pregnancy and lifestyle behaviours.

#### Preferences regarding the type of rewards and the system of allocation

##### *Preferred rewards*

The rewards most participants preferred were baby products (nine participants) such as onesies, pacifiers, hats and socks, or vouchers for products such as books, sportswear, pregnancy vitamins, luxury goods, and healthy food (all participants.) Four participants also stated that it would be painful to receive baby products if becoming pregnant would turn out to be difficult for them. *“Yes, I think so too. That [baby products] can be very hurtful, yes. Because you have all those nice baby things at home, but that which you really wish for, has not yet happened. You can't make that baby appear in your womb. I think it can be very hard if it doesn’t… If it takes a long time.” (Interview L, 31 y.o., one child.)*

##### *Preferred reward system*

We described two forms of reward systems: System 1; the user can save points by, for example, abstaining from smoking. For each day of continued abstinence, the user earns a certain amount of points which can be saved or used in the web shop. The user has the liberty to choose between small but frequent rewards or save points for a big reward. System 2: the user chooses a goal, like smoking cessation, and commits to a specific timeframe in which not to smoke. After this timeframe, the user chooses one of three suggested rewards.

Five participants indicated that they prefer a system in which you can save points continuously and can choose when and on what to spend those points. The main reasons behind this were having the freedom of choice and feeling seriously demotivated if they lost all progression due to one mistake or relapse. Three participants disliked the idea of saving points continuously, which, in their view, would lack an endpoint. Four participants did not have a preference. *“I would find it more attractive if I had multiple choices. – I would like to choose my reward, you know? Maybe, after a while, you don’t want that specific thing anymore, or you get it from someone else. Know what I mean?” (Interview J, 33 y.o., three children.) “For example, if I start smoking again after the third week, I would be very annoyed because I did not yet reach my goal of four weeks. And then I would have to start all over again.” (Interview K, 22 y.o., nulliparous.)*

#### Barriers

##### *Anticipated barriers*

During the interviews, we have identified three barriers that may prevent the use of an app-based nudge for pregnancy preparation: (1) asking payment for the app, or (2) women having to contact a healthcare provider to access the app, and (3) women not considering themselves as part of a target group for preconception care. Five participants stated that having to pay for the app would prevent them from using it, even if it were a small amount like €0.01,-.* “Well, I would not download the app [if I had to pay for it], because I just don’t buy apps. Not that I’m against it, but I just don’t do that.” (Interview I, 19 y.o., nulliparous.)*

As an alternative, we asked if they would mind calling or e-mailing a healthcare provider, for example, a general practitioner or midwife, to provide them with a code to access the app. Most participants had no objections, but three participants said contacting a healthcare provider would cost them too much effort, invade their privacy or make trying to conceive “too real”. *“I don’t know… I think [asking a general practitioner for a code] would make it too intrusive. Interfering in private matters.” (Interview J, 33 y.o., three children.)*

Women not considering themselves to be part of the target group for PCC, may form a barrier that prevents them from participating. *“I don’t smoke, but I do drink [alcohol], so yes… But those are things that you are not going to quit 3 months in advance – I don’t know, I just quit after I found out I was pregnant. I didn’t really feel like I had to quit in advance.” (Interview J, 33 y.o., three children.)*

A fourth barrier was identified in advance of this study and concerned not being able to reach women of the target group and promote the use of the app. We discussed this barrier with the participants and asked them how we could best reach them. Eleven participants said that distribution through social media would be ideal. Facebook was mentioned most often, closely followed by Instagram, Snap Chat, YouTube, and other (semi-social) media forms such as websites for parents (to-be) and influencers. Facebook was deemed most fitting for this purpose due to the possibility of personalised adds in ‘mommy groups’ and its widespread use amongst our participants and their friends. One participant stated that she would rather be informed of the app in a more intimate way. For example, by a GP or when visiting a pregnancy fair. “*It seems like a really good idea to advertise through Facebook. I think Facebook is one of the socials that is really personal. Almost everyone I know, both young and old, uses Facebook. I would recommend using Facebook for advertising. Especially if you create a page for the app.” (Interview K, 22 y.o.)*

To investigate if shame or stigmatisation would be a barrier, we specifically asked the participants if they would feel ashamed of using the app or expect others to look down on them. No participants expected either such thing. *“I think people would say “well, that's a nice system. So, you are preparing for pregnancy and you can earn rewards!””. I think a lot of people would just see it as extra motivation. The same thing happens with smoking cessation. No one says “Oh you need an app to quit smoking?”!” (Interview F, 42 y.o., one child.)*

## Discussion

We examined the preferences and opinions of vulnerable women towards a nudge in the form of an app that aims to help them adopt healthy lifestyle behaviours preconceptionally by offering rewards.

### Usefulness of an app as integral information source

Overall, the participants deemed an app as integral source of information useful. They stated that currently, the amount of information could sometimes be overwhelming and scattered. In order to prevent adding even more scattered information, the information in the app should be all encompassing, preferably addressing women’s journey from the preconception period up until the end of the postpartum period.

### Permissibility & effectiveness

Although two participants expressed some hesitance at first, all deemed offering rewards for adopting healthy lifestyle behaviours permissible. This is the result of participants making a clear distinction between already present intrinsic motivation and added extrinsic motivation, stating that the rewards would increase the latter, and thereby increase the overall motivation for behavioural change. They viewed receiving rewards as permissible mainly because all mothers-to-be would first and foremost be intrinsically motivated regardless of the intervention. In other words, the responsibility for preparing for pregnancy lies with the mothers-to-be. However, they also think that it would be a good thing if women were offered support by their caregivers through a loyalty program.

As stated in the results above, participants mentioned that women who would be interested in using the app, already have a certain amount of (intrinsic) motivation to make lifestyle changes in preparation for pregnancy. Participants expected the rewards for healthy lifestyle behaviours to tip the balance towards implementing and maintaining these new behaviours. The consensus was that offering rewards would be effective in encouraging women to adopt healthy lifestyle behaviours and may lead women towards information on pregnancy preparation which they otherwise may not have searched for. Increasing women’s knowledge on how to get pregnant healthily makes them more aware of their own influence on the matter. This awareness will allow them to actively make a choice (whether or not) to prepare for pregnancy. This strengthens their ability to set, and strive for goals that they themselves value. In other words, it will empower them which is in line with the duty to respect autonomy [19]. Furthermore, it also aligns with the principle of beneficence as it will likely lead to better pregnancy outcomes [19, 26].

### Content

These vulnerable women appreciate an app for the support of pregnancy preparation that includes information on healthy behaviours such as nutrition, folic acid supplement use, exercise, and the effects of abstaining from alcohol and tobacco. In accordance with the findings of Ismaili M’hamdi et al. [12], we found that the participants seemed to find getting pregnant as soon as possible of the highest importance. Therefore, the app should include a straightforward section on female anatomy, menstrual cycles, and how to time intercourse for a pregnancy.

Some participants mentioned that they would be more willing to change their lifestyle if they did not get pregnant quickly. Therefore, this section should also include information regarding the preconception period and the fact that a healthy lifestyle increases the chance of becoming pregnant. However, to prevent harm, it is of paramount importance to impress upon the users of the app, that getting pregnant relies on a multitude of factors, of which not all can be influenced by themselves. Being made to feel guilty or responsible for not getting pregnant quickly would be in conflict with the principle of non-maleficence [19].

Some participants mentioned that they would be more willing to adhere to certain advice if they knew the rationale behind it. For example, not just stating that folic acid supplement use is important for the health of the future baby, but also explaining that it prevents neural tube defects such as spina bifida or a cleft lip. Since it is well known that health literacy may be low in the target group of vulnerable women [27], a certain balance must be found between explaining the background of the guidelines and keeping the information straightforward and accessible. For example, showing women an animation on smoking in pregnancy may be a more effective way of information transfer than asking them to read a text. Optimising the understanding of information by the target group, is a form of respecting autonomy as it supports making informed choices about preparing for pregnancy (or not).

### Type of rewards and system of allocation

By definition, nudging should not significantly change economic incentives. Giving out rewards with a high monetary value may coerce women into using the app, which is in conflict with the duty to respect autonomy and possibly in conflict with the principle of non-maleficence [19]. The rewards preferred by the participants of this study are, indeed, of limited value. For instance, baby products such as pacifiers and onesies were mentioned multiple times, in contrast to expensive options such as baby furniture which was not mentioned once. It is likely that participants only mentioned small rewards that they feel would motivate them, but not make them feel uneasy or forced.

We have proposed two different systems of allocation to our participants. Both systems function as a *‘convenience’* nudge [28], as they lower the threshold for preparing for pregnancy, by offering rewards and making it more fun.

System 1, in which women collect points continuously and spend them at their own discretion, is an example of a token economy [29]. Token economies rely on three pillars: Tokens, back-up reinforcers and target behaviours. The tokens (i.e. the points) have no value, other than being exchangeable for back-up reinforcers(i.e. rewards). The strength of this system lies in the immediate reinforcement of earning points and the possibility of earning a smaller amount of points for partially choosing healthy behaviour. In other words, the participants will be reinforced throughout the process of preparing for pregnancy and not only for doing it perfectly. Furthermore, the option to save points and spend them at a later time on a more valuable reward (delayed gratification) also triggers practicing self-control, which is an important skill to develop when preparing for pregnancy.

System 2, in which women choose a goal and commit to a certain behaviour in a specific timeframe, works differently. In addition to being a *‘convenience’* nudge, it also falls under the category of *‘precommitment strategy’ *[28]. Precommitment strategy entails committing to behaviour that aligns with a long-term goal, and restricting other options that do not align with the long-term goal, but, in the meantime, may become desirable. For example, in the case of smoking cessation, nicotine cravings may cause the participant to smoke a cigarette despite wishing to quit smoking. In other words, the long-term goal of smoking cessation is in conflict with the short term goal of avoiding unpleasant cravings. System 2 tries to prevent smoking by increasing its costs. If, for instance, a participant has committed to not smoking for four weeks, lighting a cigarette two weeks in will immediately reduce cravings, but also ‘robs’ her of the reward for which she has already endured two weeks of unpleasant cravings. Choosing to invest more, purely based on the fact that one has already invested a lot, is known as honouring sunk costs [30]. Although, in this case, honouring past costs would be a more fitting term.

A small majority of participants preferred the system in which they collect points and spend them at their own discretion. From an ethical point of view, this is indeed a prudent option. Some women may prefer to receive small but frequent rewards, while others may prefer saving points in order to receive a more valuable reward at a later time. Allowing participants to choose freely will lead to selection of timing and rewards that encourage them the most. The freedom to choose is in line with the duty to respect autonomy and, at the same time, provides maximum encouragement which adheres to the principle of beneficence. Moreover, it also aligns with the principle of justice due to optimisation of cost-effectiveness, and the principle of non-maleficence, as choosing freely will prevent painful situations in which women receive rewards that may trigger negative feelings, like receiving baby products when the participant is not pregnant yet.

### Barriers

During the interviews, three barriers to the use of the app and nudge were identified. The first two concerned barriers linked to the distribution of the app. To prevent use of the app by, for example, women who do not wish to conceive but do want rewards, we proposed the options of payment or having to contact a healthcare provider to access the app. Multiple participants felt that both these options would form a significant barrier that would prevent the use of the app.

Paying or asking your healthcare provider for an app that helps you to prepare for pregnancy requires a certain amount of commitment and compliance from the user [31]. This may form a barrier for women who are not convinced they need support or who do not wish to actively prepare for pregnancy. Furthermore, some women rather not share their wish to have a child with their general practitioner, viewing it as too private to discuss or as a natural process that should not be medicalised [32].

Nevertheless, if you want to specifically target a certain group, some form of selection or registration must take place. For example, the app can be offered by general practitioners to women they consider vulnerable for adverse pregnancy outcomes if they visit the practice for other reasons than becoming pregnant. Ismaili M’hamdi et al. [12] have shown before that women do not mind being informed about pregnancy preparation in relevant situations, like when they are prescribed teratogenic medication. Considering the effect on pregnancy outcomes, it would be prudent for healthcare providers to utilize every opportunity that arises to discuss pregnancy preparation. Especially within primary care, links from, for example, chronic diseases to pregnancy preparation are easily made. However, this valuable opportunity to discuss pregnancy preparation is often missed [33].

The third barrier consists of the eligible women not being motivated to start with the app. Women do not often consider themselves to be part of the target group for preconception care and may not be aware of their risk of adverse pregnancy outcomes [34, 35]. This may cause them to not be interested in using the app. Of course, lack of interest in the app could also be the result of a personal choice not to prepare for pregnancy at all or in this specific way. One participant in this study stated that she would not use the app as she likes to follow her own rules, such as not abstaining from alcohol before a positive pregnancy test. It is our hope that the rewards will nevertheless convince women to partake in PCC regardless of their initial attitude towards PCC.

We have researched participant’s perceived likeliness of stigmatisation as a possible barrier by asking if they would feel ashamed of using or ‘needing’ an app-based nudge to prepare for pregnancy or would expect others to look down upon them for using such an app. The participants stated that they would be very open about it, recommending it to their friends and family, not expecting any judgement or stigmatisation at all. This is very important because stigmatisation could lead to possible harm and is therefore in conflict with the principle of non-maleficence. If it was widely known that the app specifically targets women who are vulnerable for adverse pregnancy outcomes, it could change the way participants, and the people around them, feel about it. Therefore, it should not be disclosed that the app, at least in the beginning, specifically targets vulnerable women.

Since this specific target group is hard to reach and does not easily participate in PCC, we asked our participants in which ways we could best reach them. Using social media and specifically creating a Facebook page and personal adds was mentioned most often. Furthermore, participants thought that the possibility of earning rewards would lead to an increased interest in the app amongst their peers. Enthusiastic users might lead to increased use of the app in the target group due to ‘word of mouth distribution’. Therefore,’word of mouth distribution’ should be included in future implementation strategy for the app [36].

### Influencing choice behaviour through the app

If we develop the app according to the participants’ wishes, it will influence choice behaviour in multiple ways, by using multiple types of nudges. One could state that the app itself would be the macro-level nudge that houses other micro-level nudges.

The app, as macro-level nudge, belongs to the category of *‘warning nudges’* because it tries to warn women, in a positive way, that they need to take action to prevent adverse pregnancy outcomes [28]. Giving out rewards to encourage pregnancy preparation and PCC uptake could be considered the main micro-level nudge of the app. The game like element of saving points and earning rewards is considered a *‘convenience’* nudge because it lowers the threshold of preparing for pregnancy, by making it more fun. Other micro-level nudges are, for instance, the possibility to talk to peers within the app, which may trigger healthy behaviour through *‘use of social norms’*. *‘Reminding’* women to make healthy choices is also considered a nudge as it puts relevant information into focus. Offering information in a straight forward, simplified manner is considered a *‘simplification’* or even a *‘convenience’* nudge because it facilitates successful transfer of information. Even the smallest of nudges may add to a positive effect on preparing for pregnancy. For example, offering an increasing number of points for log-in streaks may increase daily use of the app, leading to more exposure to the other micro-level nudges.

As the app contains many different kinds of nudges, it is likely that some effects regarding pregnancy preparation and PCC uptake could be expected, even if points were not exchangeable for rewards. For example, women could be offered the option of sharing the amount of points they have earned to compete with other women for top rankings. For now, however, we expect the promise of earning rewards to play a large role in initial recruitment of participants. Therefore, offering the app without rewards may be explored in the future.

### Use of the app in perspective

Countering adverse pregnancy outcomes in women who are particularly vulnerable for them, is a greatly complex matter that requires prevention, intervention and durable changes in social policy. Even if using an app-based nudge turns out to be effective in reducing adverse pregnancy outcomes, it is still of utmost importance to keep in view that these outcomes, within our target group, are first and foremost the result of their unfavourable, unfair, social circumstances. We will always advise to use an intervention such as this app, in addition to a wider range of interventions, aimed at ameliorating the social circumstances of these women in a durable, lasting way.

### Strengths and limitations

This study provides unique insights into the preferences and opinions of vulnerable women regarding an app-based nudge. Including the target group in the developmental process to this extent, will allow us to cocreate an app-based nudge that is tailor made to their needs. We have identified four limitations in our study.

First, possible selection bias with regard to women’s willingness to apply for an interview study. The women who have applied for this study may not be part of the most vulnerable group as it is known that vulnerable women are often hard to reach. Second, both women with an intermediate and a low educational level have been included which has led to some heterogeneity within our study population. Third, the small sample size of twelve participants limits stratified analysis. Fourth, our research was of an exploratory nature and we therefore used semi-structured interviews. More research is necessary to affirm our findings and we recommend future researchers to use a scoring system to quantify the qualitative data.

### Future research

Currently, we are in the process of developing the app. In the next phase, we will test the usability and feasibility of the app in a pilot study. If the results of the pilot study are satisfactory, we will conduct a cohort study to determine the effectiveness of the app in supporting pregnancy preparation.

When we include participants for these studies, we may again encounter selection bias and educational level heterogeneity. In an effort to avoid this, we will actively stimulate ‘word of mouth’ distribution by offering a small reward to invite a friend, offer the app through healthcare practices and programs that work with vulnerable women and contact community centres and schools in low income neighbourhoods to try to include women we, otherwise, would not reach. If, with these efforts, we still not reach and include the most vulnerable women, we will focus on the intermediate group who still comprise an important part of our target group.

If the app proves effective in encouraging women to prepare for pregnancy and visit a preconception care consultation, we hope to distribute it nationwide and help reduce adverse pregnancy outcomes. This reduction in adverse outcomes and their costs, could convince healthcare insurance companies to include the app in their reimbursements, as is the case for the lifestyle coaching app Smarter Pregnancy, which costs €30 ($33). We expect that €30,- will be enough to cover the expenses of the app and the preferred rewards.

## Conclusion

Our study examined the preferences and opinions of vulnerable women towards an app-based nudge that helps them adopt healthy lifestyle behaviours to adequately prepare for pregnancy. Through this app, the target group will be nudged with rewards to encourage them to improve their health and pregnancy outcomes through adoption of healthy lifestyle behaviours.

To develop an effective and morally permissible nudge, the preferences of the target group are of paramount importance, as they often know well what they find permissible and what works for them. Based on our results, we recommend an app that is easy to download and easy to use. The information in the app should be straightforward and complete, and provide the target group with one all-encompassing source of reliable information. Women should be able to collect and spend the points they earned in a clear and simple fashion. The rewards participants can purchase with these points should be diverse, so that there are plenty of options to choose from at different times (and level of points).

Special attention must be paid on how to implement this intervention for women with a low SES as they are a difficult group to reach and motivate to participate in PCC. Using social media and personalised adds seems ideal. Additionally,’word of mouth’ distribution could prove to be an effective implementation strategy. 

## Data Availability

The datasets used and/or analysed during the current study are available from the corresponding author on reasonable request.

## Abbreviations

SES: Socioeconomic status
PCC: Preconception care
mHealth: Mobile Health
App: Application
